# Codon usage bias and the evolution of influenza A viruses. Codon Usage Biases of Influenza Virus

**DOI:** 10.1186/1471-2148-10-253

**Published:** 2010-08-19

**Authors:** Emily HM Wong, David K Smith, Raul Rabadan, Malik Peiris, Leo LM Poon

**Affiliations:** 1 Department of Microbiology, The University of Hong Kong, Pokfulam, Hong Kong, China; 2 Department of Biochemistry, The University of Hong Kong, Pokfulam, Hong Kong, China; 3 Department of Biomedical Informatics and Center for Computational Biology and Bioinformatics, Columbia University College of Physicians and Surgeons, New York, USA

## Abstract

**Background:**

The influenza A virus is an important infectious cause of morbidity and mortality in humans and was responsible for 3 pandemics in the 20^th ^century. As the replication of the influenza virus is based on its host's machinery, codon usage of its viral genes might be subject to host selection pressures, especially after interspecies transmission. A better understanding of viral evolution and host adaptive responses might help control this disease.

**Results:**

Relative Synonymous Codon Usage (RSCU) values of the genes from segment 1 to segment 6 of avian and human influenza viruses, including pandemic H1N1, were studied via Correspondence Analysis (CA). The codon usage patterns of seasonal human influenza viruses were distinct among their subtypes and different from those of avian viruses. Newly isolated viruses could be added to the CA results, creating a tool to investigate the host origin and evolution of viral genes. It was found that the 1918 pandemic H1N1 virus contained genes with mammalian-like viral codon usage patterns, indicating that the introduction of this virus to humans was not through *in toto *transfer of an avian influenza virus.

Many human viral genes had directional changes in codon usage over time of viral isolation, indicating the effect of host selection pressures. These changes reduced the overall GC content and the usage of G at the third codon position in the viral genome. Limited evidence of translational selection pressure was found in a few viral genes.

**Conclusions:**

Codon usage patterns from CA allowed identification of host origin and evolutionary trends in influenza viruses, providing an alternative method and a tool to understand the evolution of influenza viruses. Human influenza viruses are subject to selection pressure on codon usage which might assist in understanding the characteristics of newly emerging viruses.

## Background

Influenza has been one of the most important infectious diseases of humans. It poses a threat to health and causes significant negative economic impacts on society every year. The last century saw 3 influenza A pandemics: H1N1 in 1918, H2N2 in 1957 and H3N2 in 1968 [1,2]. Since 1997 avian H5N1 influenza has been infecting humans zoonotically resulting in a high mortality rate [3] and there were fears it might cause the first pandemic of this century. However, the influenza pandemic of 2009 was caused by an H1N1 multiple reassortant with genes derived from viruses that originally circulated in the swine, avian and human populations [4,5].

In order to evade the host immune response, human seasonal influenza virus changes its antigenicity by introducing novel mutations in its surface proteins (called antigenic drift) [6]. The influenza pandemics in the last century were caused through antigenic shift, which occurs when there is a reassortment of the surface protein segments between viruses, resulting in a virus that was immunologically novel to humans [7]. It has been observed that the influenza virus is subject to host immune selection pressure and undergoes rapid evolution in the antigenic regions, especially when the virus crosses the host species barrier [8]. To better prepare for future pandemics, a detailed understanding of the basic biology of this virus, especially its evolution and methods for host adaptation, is needed.

The genetic code is degenerate and synonymous codons, those that code for the same amino acid, have been observed to be used unequally in most species [9-14]. This uneven codon usage was not neutral as some had suggested, but related to gene expression [9,14-16], nucleotide usage [17], protein structure formation [18-20], and even viral RNA packaging [21,22]. Two major models have been proposed to explain codon usage, the translation related (or selective) model and the mutational (or neutral) model.

In the translation related model, one postulate is that there is a co-adaptation of synonymous codon usage and tRNA abundance to optimize translational efficiency. A correlation between codon usage and gene expression is expected [23]. This is seen in *Saccharomyces cerevisiae *[24] and might be due to intrinsic codon preferences reflecting the stability of codon-anticodon interactions [9]. In Epstein-Barr virus latent stage genes appear to deoptimize codon usage perhaps to reduce competition with host cell translation [25] and papillomavirus codon usage appears optimized for expression in certain cell types [26]. Attenuation of polio virus activity was achieved by reduced translation for viral genes constructed with disfavored synonymous codon pairs [27]. An alternative translation related postulate is the possibility of fine-tuning the kinetics of protein translation by a combination of rare and common codons [28]. This has been demonstrated for the hepatitis A virus capsid protein [29,30].

The mutational model postulates that genetic compositional constraints influence the probability of mutational fixation [23] and this has been found in many species [12,13,15,31,32]. In particular, the influence on codon usage of reduced CpG dinucleotide content might be related to anti-viral responses by the cell [33,34]. It should be noted, however, that the models are not mutually exclusive [23].

The replication cycle of the influenza virus depends on host machinery and the virus utilises host cellular components for its protein synthesis. Therefore codon usage in this virus and its hosts could be expected to affect viral replication. Although some studies have been performed on the general codon usage of influenza [35-37], little has been done to investigate the effect of selection pressure imposed by the human host on the codon usage of human influenza viruses and trends in viral codon usage over time.

Codon usage of mammalian and avian influenza viruses was examined in this study using relative synonymous codon usage (RSCU) values [38] and Correspondence Analysis (CA) [39]. These techniques are well established in studies of codon usage [37,40,41]. If influenza viruses and their subtypes that circulate in different hosts have different codon usage biases, it should be possible to identify the subtype and host source of a virus using this property. Host selection pressures, if any, that affect codon usage in influenza viruses might be identified by this approach.

Codon usage bias, which is largely determined by the nucleotide in the third codon position, allows a different perspective on viral evolution to be examined. In phylogentic studies, especially involving distantly related sequences, this codon position may be discarded due to possible saturation of mutations at the position. Phylogenetic analyses of large numbers of sequences require distance-based methods which reduce the comparison of sequences to a single distance value. Codon usage studies retain some of the underlying structure of the coding sequences in the comparison and may give another perspective on evolutionary changes.

Correspondence analysis was primarily used to analyse codon usage in influenza and host sequences and the resulting patterns were visualized by projection onto 3-dimensional graphs. It was possible to separate viruses by their host, and also by subtype for human viruses, using this technique. The consistency of the findings from this technique with host specificity and viral subtype allowed a general tool to be created to analyze newly emerged influenza viruses. This approach also provided an opportunity to assess the origin of viral strains, such as 1918 H1N1. Changes in codon usage with the time of virus isolation were observed for human influenza viruses so that the translational related and mutational models of codon usage bias could be examined.

## Results

RSCU values of the 59 relevant codons were determined for all the sequences studied in this work. To provide a way to analyse and visualize these data, CA was used on the RSCU values of different sets of viral and host sequences. For large multi-dimensional datasets, CA allows a reduction in the dimensionality of the data so that efficient visualization that captures most of the variation can occur [39]. Here, the first 3 axes from the CA analyses were used to provide 3-dimensional visualization of the relationships among the sequences. A further dimension of color was used to identify sequences with different features (e.g. viral host, subtype and year of isolation) in an analysis.

### Avian and human influenza virus codon usage

**Figure 1:**
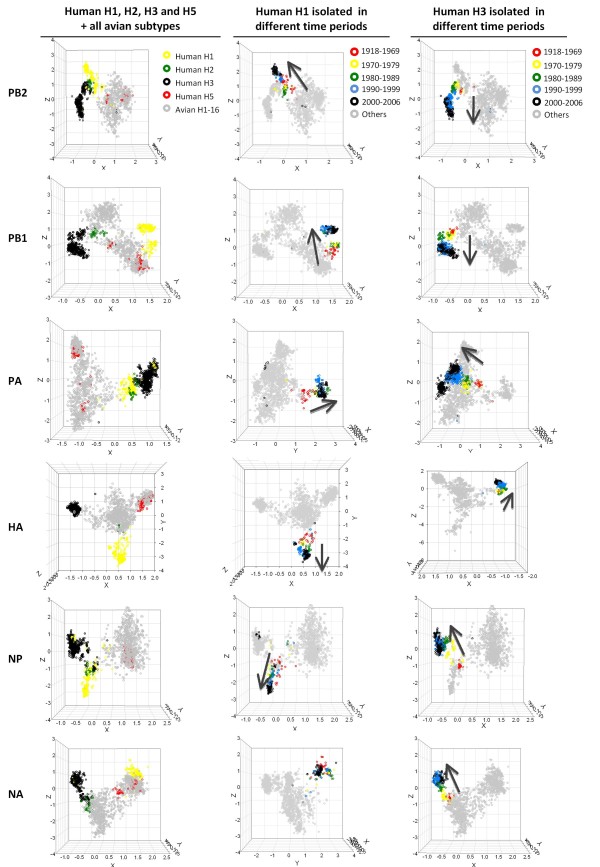
**CA of human (seasonal H1-H3 and H5) and avian influenza viruses**. Each viral gene is displayed in a 3-dimensional representation. The X, Y and Z axes are in arbitrary scales generated by the CA and the weight of each codon in these axes varies in different segments. (A) Human influenza colored by subtype (B) seasonal human H1 subtype colored by year of isolation and (C) seasonal human H3 subtype colored by year of isolation. The codon usage trends with time of viral isolation are indicated by arrows. The orientations of NA of H1 and HA of H3 in the graphs were altered for better presentation.

Projection of avian and human influenza virus codon usage, by gene segment, onto the first 3 axes after CA revealed that the viruses for the different hosts have differing codon usage biases (Figure 1A). Human host virus subtypes are indicated by color and, apart from the human H5N1 genes and some genes from human H2N2, the host groups are well separated from each other. For some of the human viral genes (PB2, PA and NP) a single human viral subtype cluster is formed but for the others (PB1, HA and NA) the subtype clusters are separated (Figure 1A). The topology of these clusters is consistent with the natural history of these human viruses (see below and Discussion).

Human H5N1 genes, which are of avian origin, are found in the avian virus codon usage cluster as would be expected. Three genes from human H2N2 influenza viruses (PB1, HA, NA), also of avian origin, are located in the avian virus cluster. However, the avian origin genes of human H3N2 (HA and PB1) extend from the avian cluster. Some human H1 viral genes (e.g. PA) are found in the human H3 cluster. When genes from avian virus subtypes were examined they tended to form a single large cluster (Additional file 1). Genes from the more recently emerged avian H5N1 subtype showed a more distinct subtype cluster and the HA gene showed distinct subtype groupings.

### Year of isolation of human H1 and H3 viruses

Codon usage in both human H1 and H3 viral genes could be examined according to the year of virus isolation with this CA result. By coloring the H1 and H3 virus subtypes by year of origin (Figure 1B, H1; Figure 1C, H3), a trend of change in codon usage with time could be seen for both viral subtypes. This increased the separation of the human viruses from the avian viruses, and the trends in codon usage of the two human subtypes were not convergent. No trend in codon usage with year was observed for avian influenza viral subtypes (data not shown).

### Codon Usage Outliers

A small number of sequences from each host or subtype in the codon usage plots (Figure 1 and Additional file 1) were observed to be closer to, or within, the cluster of the other host or a different subtype group of their host. These were denoted as codon usage 'outliers'. Some representative examples and their descriptions are presented in Additional Files 2 and 3). On further examination, most of these outlier sequences were found to be generated from zoonotic transmissions or reassortments between viruses of different origin. For example, the PB2 gene of human A/Victoria/1968 (H3N2) was found to be of human H1 origin (sequence ID 1623, Additional files 2, PB2).

### Use and validation of CA derived axes as a tool for virus identification

Viral sequences that were not used as part of the CA analysis can be placed in the graph of the CA results by taking the cross product of the relative RSCU vectors of those sequences and each of the first 3 eigenvectors (i.e. those that formed the X, Y and Z axes in the CA visualization) (Additional file 4). Using this formula, the positions of new viral sequences in these graphs were estimated. Thus the CA created a tool to determine the relationship between a novel sequence and those used in the CA without the need for extensive sequence gathering and mathematical re-calculations.

Cross-validation by randomly assigning sequences to 5 equal groups was used to verify this strategy. CA was performed on 80% of the sequences and the remaining 20% were predicted by applying the above formula for the 5 groups. The clear similarity of the total and a representative cross-validation analysis are shown (Additional File 5). A second test analyzed sequences of human influenza viruses that emerged from 2007 to 2009 by this approach and inserted their scores into the existing CA graphs. As a control, CA was performed on the extended dataset containing the new sequences. The topologies of the sequences from the original and extended datasets were similar (Additional file 6).

### Relationship of pandemic H1N1/2009, H1N1/1918 and canine H3N8 to other influenza viruses

**Figure 2:**
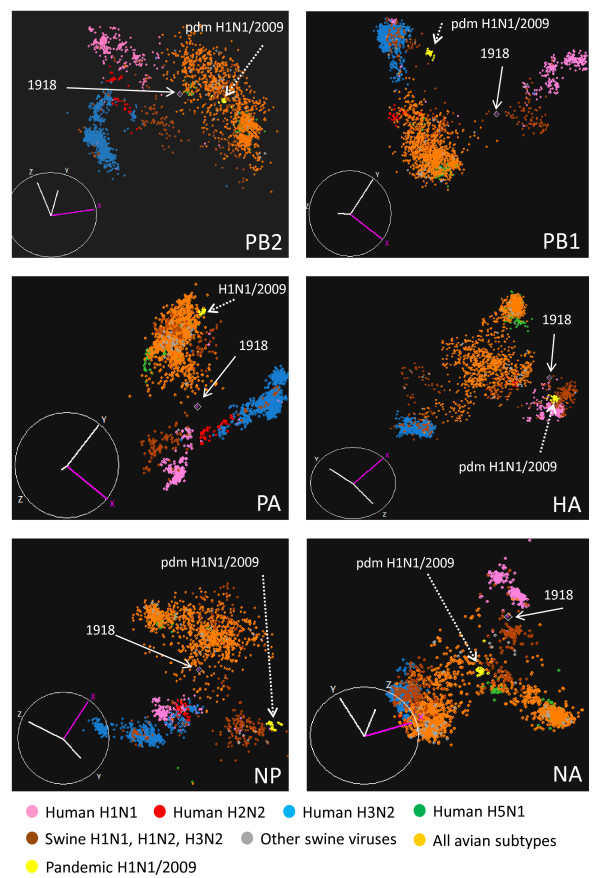
**CA of human (seasonal H1-H3, H5 and pandemic H1/09), avian and swine influenza viruses**. The viral hosts are differentiated by color. Viral genes derived from A/Brevig Mission/1/1918 (1918) and pandemic H1N1/2009 (pdm H1N1/2009) are indicated by arrows. The X, Y and Z axes are codon usage and are in arbitrary scales generated by the CA for each segment.

CA was performed on the codon usage of seasonal human, avian and swine influenza viral sequences, together with the recent pandemic human H1N1/09 viral sequences. As shown in Figure 2, the pandemic H1N1/09 virus was found to have avian codon usage patterns for PB2, PA and NA, human/human-like swine H3 patterns for PB1 and a classical swine H1N1 pattern for HA and NP (Figure 2, yellow circles). Many swine H1N2 and H3N2 triple reassortant viruses are located close to the pandemic H1N1 virus.

This clustering method was also applied to investigate the possible gene sources of 1918 pandemic H1N1 using the A/Brevig Mission/1/1918 strain as the reference sequence. The PB1, HA and NA genes of 1918 H1N1 were found to be located close to mammalian H1 influenza viruses in the CA (Figure 2). Codon usage patterns similar to those of avian influenza viruses were observed for the PB2 and NP genes of A/Brevig Mission/1/1918, while its PA gene was located in the interface between the avian and mammalian clusters (Figure 2). The ten sequences that had the shortest distance from each of the genes of A/Brevig Mission/1/1918 are summarized in Additional file 7.

The emergence of canine H3N8 virus has been well-documented as an *in toto *transfer of equine influenza virus [42]. CA of the codon usage of human, avian, swine, canine and equine influenza viral sequences located the canine H3N8 viruses in the equine viral cluster (Additional file 8).

### Codon usage of influenza viruses and their hosts

As influenza viruses that infect different hosts have different codon usage biases (Figure 1), codon usage in influenza viruses and their hosts was examined. Average RSCU values of influenza virus subtypes and their hosts were calculated (Additional file 9). Eight codons, all of which contain the dinucleotide CpG, were under-represented in both human and avian influenza viruses (TCG, ACG, GCG, CCG, CGC, CGA, CGG and CGT; RSCU value <0.6). Nine codons (TCG, ACG, GCG, CCG, GTA, TTA, CTA, ATA and CAA) were under-represented in all the viral hosts (RSCU ≤ 0.62). Except for CAA, all these codons contained either CpG or TpA at their 3' ends. The under-representation of CpG or TpA dinucleotides has been reported in many living organisms [43-45], however, only CpG was under-represented in the influenza viral genomes.

Five codons, which were purine rich, (ACA, GCA, AGA, AGG and GGA) were over-represented in all the viral genomes (RSCU > 1.6). A similar over-representation of codons was only found for CTG and GTG in the hosts. The most commonly used synonymous codons were the same within the viral or host groups (except for 1 amino acid in the viruses and 4 amino acids in the hosts) but were different between the influenza genomes and their hosts for 14 of the 18 amino acids (Additional file 9, highlighted in bold).

### Codon usage trends in human influenza viruses

**Figure 3:**
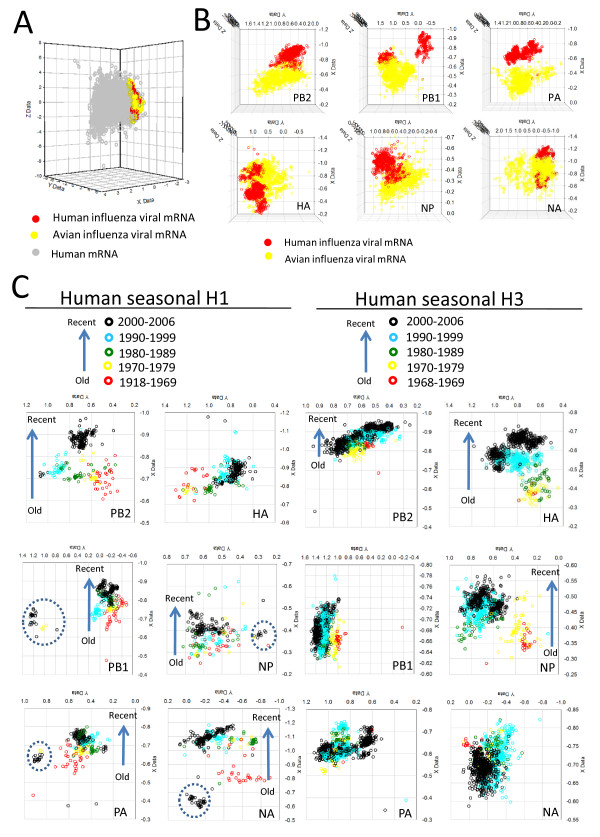
**Condon usage of influenza virus in the context of human coding sequences**. RSCU values of all six human and avian viral segments and human RefSeq mRNA sequences were subjected to a joint CA. (A) CA of all influenza viral and human mRNA. (B) and (C) are subsets of the data shown in (A). (B) Human and avian influenza viral datasets by gene extracted from panel A. (C) Seasonal human H1 (left) and seasonal human H3 (right) datasets extracted from panel A. The unidirectional trend on the X coordinates found in the H1 (PB2, PB1, PA, NP and NA) and H3 (PB2, HA, NP) genes is indicated by an arrow. Examples of outliers (e.g. H1N2) are marked by broken blue circles.

To better understand the change in codon usage over time that was seen in human influenza viruses (Figure 1), a CA analysis of all 6 viral genes in the context of human RefSeq coding sequences was performed. The human genes formed a cluster separate from those of the viruses which were again separated based on host type (Figure 3A). Individual human viral genes were also well separated from their avian counterparts (Figure 3B) as expected from the initial analysis. Human H1 and H3 viral subtype genes were extracted from the graph and examined separately, while still within the context of the analysis with human genes. Generally the genes had different trends in codon usage change and in different overall directions (Figure 3C). Many of these human viral genes had a unidirectional trend on the X-axis of the combined host and viral CA (Figure 3C, highlighted by arrows) which was more prominent for some genes (e.g. H1 PB2 and H3 HA). Outlier groups, as in the earlier analysis of the viral sequences, were also found (blue dotted circles in Figure 3C).

To examine whether a general trend in the codon usage of seasonal influenza viruses exists, a correlation analysis of the RSCU values for codons of each viral gene versus the year the virus was isolated was performed. Codons that have a strong positive (r > 0.5) or negative (r < -0.5) correlation coefficient with year of virus isolation are summarized in Additional file 10. As expected, far fewer avian than human virus codons show positive and negative trends.

### Codon usage changes in human influenza viruses and nucleotide composition

Codon usage is known to be highly influenced by nucleotide composition [31]. Codons from the human viral populations that showed negative correlation trends in RSCU with year of isolation (Additional file 10) had a significantly lower A (p = 0.01) and higher G (p = 0.02) content in the third codon position than did those of positive correlation trend codons. Overall GC content in the negatively correlated group was significantly higher than that in the positive correlation trend population (p < 0.001). It was also found that the frequency of the ApA and CpG dinucleotides were significantly lower (p = 0.049) and higher (p = 0.042), respectively, in the negative correlation trend codon populations.

Using the overall nucleotide and third codon position nucleotide composition of human influenza viral PB2, PB1, PA, HA, NP and NA genes as references, it was demonstrated that the codons with negative correlation trends with year of isolation had significantly higher GC content (p = 7 E-09) and higher G usage at the third codon position (p = 0.00016) when compared with the set of full-length gene sequences. However, significantly altered nucleotide usage in the positive correlation trend population was not observed.

The GC content and third-codon position nucleotide content of the six human viral genes had individual trends with the year of virus isolation. Many of these human viral genes had a clear tendency to reduce the overall GC nucleotide usage (Additional file 11, highlighted in red). An increase in A and decrease in G nucleotide usage at the third codon position with year of isolation was also observed in many of these viral genes (Additional File 12). This was not observed in the avian viral populations (Additional Files 11-12). Human viral segments, which were shown to have a unidirectional codon usage trend (Figure 3, PB2, PB1, PA, NP and NA of H1; PB2, HA, NP of H3), were all found to have a reduction in GC nucleotide usage with year of viral isolation (Additional file 11).

### Influence of viral gene translation on codon usage

**Figure 4:**
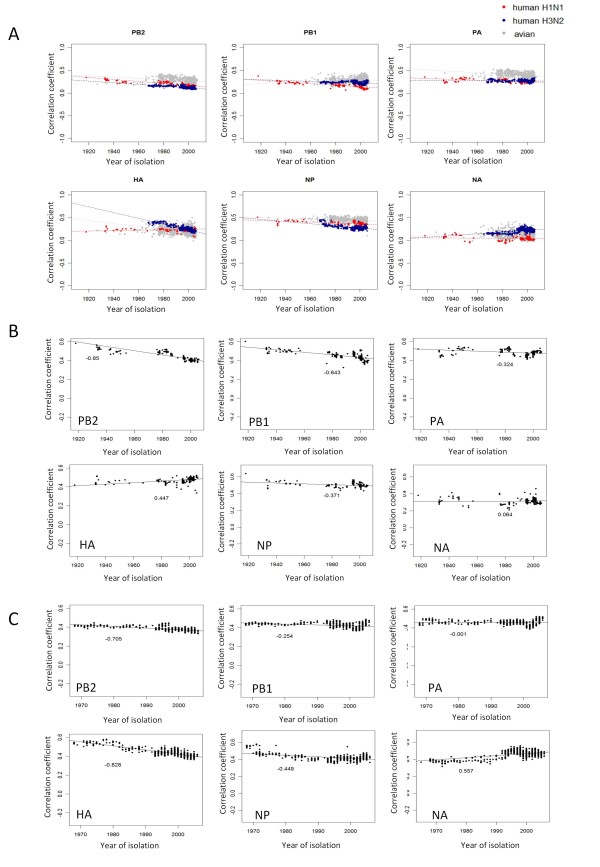
**Trends in correlation between viral and human codon usage by year of viral isolation**. (A) Correlation trend for human H1N1 and H3N2 subtypes and avian influenza. (B and C) Correlation of codon usage between H1N1 (B) or H3N2 (C) viral genes and genes expressed in human bronchial epithelial cells by year of virus isolation. The linear regression line and the correlation coefficient of each dataset are shown.

Viral codon usage (avian and human H1N1 and H3N2) by year of isolation was correlated with human codon usage (taken from the RefSeq coding sequences). With the exception of the HA (H1N1) (r = 0.27, *p *< 4e-08) and NA (H3N2) genes (r = 0.48, *p *< 2e-16), the correlation coefficients between human and viral codon usage showed a negative trend with the year of virus isolation (Figure 4A).

Human genes known to be highly expressed in human fetal lung, adult lung, adult trachea and bronchial epithelial cells [46] were used to generate 4 human tissue-specific codon usage datasets. Correlations between human tissue-specific gene codon usage and viral gene codon usage mainly showed negative or no trend with year of virus isolation (Figures 4B and 4C; Additional file 13 and data not shown). In bronchial epithelial cells (Figure 4B and 4C), codon usage in the human H1 HA or H3 NA viral genes had a positive trend with year of viral isolation.

**Figure 5:**
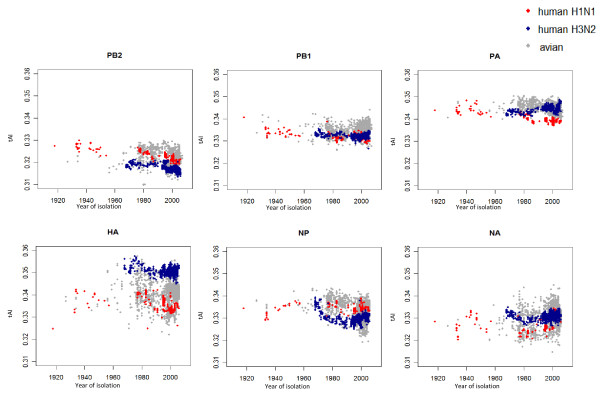
**tRNA adaptation index of influenza viruses**. The tAI of each viral gene is shown by year of virus isolation. Viral host and subtypes are indicated by color.

Human tRNA abundance could affect translation and codon usage in influenza viruses. The tRNA Adaptation Index (tAI) gives a measure of how well a gene is adapted to a tRNA pool [47]. Taking the human tRNA gene copy numbers as a reference, none of the tAI of these human viral genes showed a significant increase in tAI with year of virus isolation, except for H1 NA (r = 0.47, *p *< 2.2e-16) and H3 PA (r = 0.32, *p *< 2.2e-16) (Figure 5). Most of the human viral genes had a negative or no trend in tAI with year of viral isolation. Respiratory tissue-specific tRNA expression profiles do not appear to be available [48], preventing the application of this approach on a tissue-specific basis.

## Discussion

Codon usage bias is a distinctive characteristic of many organisms [23] and has been noted in viruses such as influenza [35-37]. As the influenza virus relies on the host cell's machinery for its replication, codon usage bias could play a role in host adaptation and the virulence of the virus. To investigate this, the 59 codons, which can display bias in their usage, were examined for influenza virus sequences of human, avian, swine, canine and equine host specificity along with human coding sequences. Relative synonymous codon usage (RSCU) was the measure of codon bias used. Dimension reduction techniques are needed to reasonably present and analyze the 59 dimensional space of codon bias, and correspondence analysis (CA) was selected to do this. For effective visualization of results, the first 3 eigenvectors were retained as axes for plots of codon usage. It should be noted that all CA studies performed here were based only on codon usage data.

Human and avian host influenza viruses isolated from 1918 to 2006 had different codon usage patterns and the human influenza subtypes (H1, H2, H3 and H5) could be distinguished based on their codon usage. Exceptions to the pattern of host separation were the human H5N1 sequences, which are of very recent avian origin, and the human H2N2 influenza PB1, HA and NA genes, consistent with their introduction from avian viral strains around 1957 [49] and the brief circulation of this virus in humans. The longer circulating H3N2 subtype genes of avian origin, PB1 and HA [49], showed a trend, over time, away from the avian codon usage cluster.

Human H1 and H3 viral subtypes have been suggested to differ in their evolutionary and epidemiological dynamics [50]. Non-convergent trends in codon usage away from the avian subtypes were observed and suggest that selection on codon usage is operating in the H1 and H3 subtypes, but not to form a common human viral codon usage pattern. The evolutionary rates of these human viral genes are not constant and can be varied across time [51]. This might also explain the nonlinear trends in human viral codon usages observed in this study.

All 6 genes of avian H5N1 viruses formed a more distinct cluster which may be due to their recent emergence and extensive surveillance over the last decade. Frequent segment reassortment events identified in other avian virus subtypes [52] resulted in their genes forming a single codon usage cluster except for the HA, and to a lesser extent the NA, genes involved in antigenic responses and so used for viral subtyping [53]. Sequences that were not located with the majority of their host group or subtype ("outliers") were, on examination, frequently found to originate from zoonotic transmission or reassortment.

CA based on RSCU values appears to be an effective tool to reveal evolutionary trends and to classify influenza sequences by host and subtype. RSCU values from a novel sequence can be mapped to the existing axes of a CA to reveal the relationship of that sequence to existing groups as shown by the validation tests performed in this work. This method also allows prompt visual identification of viral reassortants or zoonotic transfer in influenza genes without the need to perform extensive computations.

The utility of CA of codon usage to reveal evolutionary trends and relationships in influenza sequences was demonstrated by the analysis of the recent pandemic H1N1/2009 viruses and the 1918 pandemic virus. Codon usage patterns of the H1N1/2009 viruses in the CA revealed their known triple reassortant characteristics [4,5] and the locations of these genes close to many swine H1N2 and H3N2 triple reassortant viruses suggest that one of those viruses might be the precursor of H1N1. Similarly, the canine H3N8 virus was located in the equine viral cluster, reflecting its *in toto *equine origin [42].

Previously, the 1918 H1N1 pandemic was suggested to be caused by a direct zoonotic transmission of an avian virus from birds to humans [54]. However, other phylogenetic analyses of 1918 influenza viral sequences resulted in alternative hypotheses [55,56]. The CA described here indicates that the PB1, HA and NA genes of 1918 H1N1 have mammalian like codon usage, being closer to the swine viral sequences. These genes might have been introduced into the mammalian virus population well before the introduction of the PB2, and NP genes which have avian characteristics and the PA gene which has an intermediate codon usage pattern. Overall, this work supports the hypothesis that the 1918 H1N1 virus was generated by reassortment events between mammalian and avian influenza viruses [55,56].

When examined together (Fig. 3, Additional file 9), human viral genes did not have the general codon usage pattern of their host's genes. In this combined CA, trends in codon usage with the year of viral isolation were still apparent with many genes showing a unidirectional trend, to different extents, along the X axis of the combined CA. This could indicate that a common selection pressure is acting on the human viral genes alongside gene specific pressures. Many human viral codons were increasing or decreasing in their usage with time (Additional file 10), suggesting selection on codon usage at both gene and subtype levels. Avian viruses had few codons showing substantial trends in codon usage probably due to frequent segment reassortment events [52] as noted before.

Nucleotide composition is known to affect codon usage [31]. Statistically significant nucleotide differences in codons where RSCU was changing with time were found (Additional file 10) when the less used codons were compared with more used codons or with the overall sequence composition. These findings were consistent with an hypothesis that there is a host-dependent C to U mutation bias in human H1N1 viral genomes [57] and a finding that there is a selection pressure to eliminate CG dinucleotides in the viral genome [58]. No significant differences relative to overall sequence composition were found in codons increasing in usage.

This work gives further evidence for host selection pressures on human influenza acting against the use of G or C nucleotides and the use of a G nucleotide at the third codon position. The trend in codon usage patterns with year of viral isolation in the CA of Figure 3 suggests reducing GC content might be part of the selection pressure changing viral codon usage. As these mutational patterns were not observed in the avian viral genes, this indicates a human host driven process which could represent mutational pressure, fine-tuning of translational kinetics or an evasion of host cellular defences.

Selection pressure on overall GC usage and nucleotide usage at the third codon position might not necessarily both act on the same viral segments (e.g. H1N1 PA and H1N1 HA). The HA gene of the human H1N1 subtype is negatively correlated over time with the use of G at the third codon position but is uncorrelated with overall GC usage. Other selective pressure could modulate viral codon usage in a more segment-specific manner (e.g. conservation of RNA motif/structure for other virological processes, fine-tuning of translation kinetics) [21,28]. Frequent amino acid mutations of the surface proteins due to antigenic drift might also cause this effect [59].

Human genes and most viral genes had a negative correlation in codon usage over time of viral isolation. This tends to suggest that changes in viral codon usage might not affect, or might even have a negative effect on viral gene translation. Exceptions were the HA (H1N1) and NA (H3N2) genes which had highly statistically significant positive correlations. Human tissue specific codon usage might be a more relevant comparison set, however human bronchial epithelial cells, which support efficient virus replication showed the same patterns. Translational selection pressure might act on only some human influenza viral genes. Using the tAI [47] instead of human gene codon usage (tissue specific data were not available) provided similar results suggesting generally that translational pressure may not be acting. Exceptions were the H1N1 NA and H3N2 PA genes.

There is some evidence of translational pressure on the codon usage of human influenza viruses. Why this effect could only be observed in a few human viral segments is not clear. It is possible that other selective pressures on the human influenza virus are much stronger than that of translational selection thereby masking this effect. Alternatively, reduced GC content in human influenza viruses may prevent the activation of human innate immune system [60] or might cause viral mRNA to form less stable structures at lower temperatures, thereby allowing more efficient viral RNA translation in human cells [61,62] or affecting translation kinetics [28]. Further investigation will be needed to address whether translational selection has a larger role in influenza evolution.

## Conclusion

This study has shown that codon usage bias provides an additional strategy to study the evolution of human influenza viruses. By CA on RSCU values, patterns and trends in codon usage were observed that allowed different viral groups to be distinguished and evolutionary trends revealed. The effectiveness of this type of analysis was demonstrated by its ability to replicate the known evolutionary groups of influenza viruses as well as to reveal new trends. It was shown that CA of the style used here can form a valuable tool to quickly classify and identify any unusual patterns in newly isolated viruses. Application of this technique to the 1918 pandemic H1N1 provided further evidence that it is more likely to be a reassortant between avian and mammalian viruses. Continuous trends in codon usage with time of viral isolation were detected in human influenza viruses. Further analyses of codon usage suggested that viral evolution might primarily be modulated by host selection pressure on viral nucleotide content, particularly GC content. Although some evidence was found for translational related selection pressure acting on a few human influenza virus genes, the observed nucleotide compositional biases generally appeared likely to reduce the rate of viral mRNA translation. Mutational pressure, fine-tuning of translation kinetics or evasion of host anti-viral responses could be the forces shaping human influenza viral codon usage.

## Methods

### Sequence data

Coding sequences of influenza A viruses were downloaded from, the NCBI Influenza Virus Resource http://www.ncbi.nlm.nih.gov/genomes/FLU[63] and human reference coding sequences (N = 20,091) were downloaded from http://www.ncbi.nlm.nih.gov/RefSeq/. Influenza sequences were arranged in five datasets. Human and avian sequences isolated from 1918 to 2006 formed the major dataset and the other sets were: swine influenza virus sequences isolated before 2007, seasonal human influenza A viruses isolated between 2007 to 2009, novel swine-origin pandemic human H1N1 viruses isolated before 18^th ^May 2009, and canine and equine H3N8 virues.

For influenza sequences, short (<80% of the corresponding gene) and abnormal sequences were removed from the datasets, and only 6 viral genes were studied in the analysis as the short length and insufficient codon usage diversity of the other genes might bias the results. The 6 genes analysed coded for PB2, PB1, PA, HA, NP and NA, and all these genes were classified according to their viral subtypes.

### Other databases used

Codon usage data of influenza viral hosts, human (*Homo sapiens*), domestic pig (*Sus scrofa*), mallard (*Anas platyrhynchos*), goose (*Anser anser*) and chicken (*Gallus gallus*), were obtained from the codon usage database http://www.kazusa.or.jp/codon/[64]. Human tissue specific gene expression data, from a previously described human transcriptome microarray study [46], were obtained from the GEO http://www.ncbi.nlm.nih.gov/geo/[65] at accession number GDS596.

### Codon bias estimates using Relative Synonymous Codon Usage

The RSCU value of a codon [38] is its observed frequency divided by its expected frequency in the absence of usage bias (which is the average frequency of all codons for that amino acid). RSCU values are not affected by sequence length and amino acid frequency since these factors are eliminated during the computation. Codons used less than average, at average level (no bias) and more than average have RSCU values, respectively of <1, 1 and >1 [37,40,41]. Codons with RSCU value >1.6 were regarded as over-represented, while codons with RSCU values <0.6) were said to be under-represented. Stop codons and codons that uniquely code for an amino acid (ATG - methionine and TGG - tryptophan), are not relevant to an RSCU analysis. For each sequence in the datasets, RSCU values were calculated for the 59 relevant codons by a PERL script (available upon request).

### Other sequence characteristics

The tAI [66], which measures how well a gene has adapted to a tRNA gene population in terms of tRNA gene copy numbers, was estimated using CodonR [47]. Single and dinucleotide sequence composition were calculated by a PERL script (available upon request) and GC3 (GC content in the 3^rd ^base position of a codon) was computed by CodonR [47].

### Correspondence Analysis and other statistical tests

CA is a type of multivariate analysis that allows a geometrical representation of the sets of rows and columns in a dataset [39]. CA was performed on the RSCU values of the sequences studied here using the R statistical software, version 2.6.2 http://www.r-project.org and the function "corresp" from the MASS library [67]. The first three eigenvectors from each analysis were used to incorporate most information from the datasets [68] and were used as axes for visualization of the results. Sequence vectors of RSCU proportion values (codon RSCU/sum of RSCU values for that sequence) were mapped to each of these axes by the cross product of the sequence vector and the corresponding eigenvector. Graphs were plotted using SigmaPlot 10.0 (Systat Software Inc). Different colouring schemes were used to label sequences on the plots according to the different features being investigated, (e.g. host, viral subtype and year of viral isolation). Other statistical tests were performed using R http://www.r-project.org

## List of abbreviations used

CA: Correspondence Analysis; HA: Haemagglutinin; NA: Neuraminidase; NP: Nucleoprotein; PB2: Polymerase basic protein 2; PB1: Polymerase basic protein 1; PA: Polymerase acidic protein; RSCU: Relative Synonymous Codon Usage; tAI: tRNA adaptation index.

## Authors' contributions

Designed the experiments: DKS and LLMP. Performed the experiments: EHMW. Analyzed the data: EHMW, DKS and LLMP. Commented on the work: RR and JSMP. Wrote the paper: EHMW, DKS and LLMP.

## Supplementary Material

Additional file 1 **CA of human and avian influenza viruses with avian viral subtypes indicated by color**. Each viral gene is displayed in a 3 dimensional representation. The X, Y and Z axes are arbitrary scales generated by the CA. Click here for file

Additional file 2 **Outliers are enclosed by open-boxes**. Sequence numbers of outliers are indicated (see Additional file 3). Avian virus outliers are marked in red, while human virus outliers are in black. Click here for file

Additional file 3 **Descriptions of human and avian viral sequences that were marked as outliers in Additional file **2. Click here for file

Additional file 4 **Estimation of 3D coordinates of a viral sequence**. Click here for file

Additional file 5 **Cross validation of CA of PB2 sequences**. Sequences (N = 3366) were randomly assigned to 5 equal groups and CA was performed on any 4 of these dataset (i.e. 80% of the total sequences). Based on the weight generated from the train set, coordinates of the remaining 20% test dataset were predicted by applying the formula similar to the one as described in Additional file 4. Left column: Original graphs as described in Fig. 1A (Human PB2) and Additional file 1 (Avian PB2). Right column: Representative results generated from one of the test dataset. Click here for file

Additional file 6 **Comparison of the location of recent seasonal human influenza viruses in CA.** Left: CA from figure 1 with the coordinates of the recent human H1 and H3 influenza sequences (year 2007 to 2009) predicted from the eigen vectors of the original CA. Right: A CA of the combined set of sequences from Figure 1 and the recent seasonal influenza sequences. Recent seasonal influenza sequences are marked in darker color. Click here for file

Additional file 7 **The ten sequences that were closest to each of the A/Brevig Mission/1/1918 genes**. Click here for file

Additional file 8 **CA of seasonal human (H1-H3), human H5, swine, avian, canine (H3N8) and equine (H3N8) influenza viruses**. Each viral gene is displayed in a 3 dimensional representation. The X, Y and Z axes are arbitrary scales generated by the CA. Click here for file

Additional file 9 **Overall codon usage of Influenza virus types and their hosts. Under-represented codons (RSCU < 0.6) are highlighted in grey, while the most commonly used codons are in bold**. Click here for file

Additional file 10 **Codons with positive (R ≥ 0.5) and negative (R ≤ -0.5) correlations in codon usage over time of viral isolation in human H1N1, human H3N2 and avian influenza viruses**. Click here for file

Additional file 11 **Correlation coefficient (R) between viral GC content and year of virus isolation**. Click here for file

Additional file 12 **Correlation coefficient (R) between nucleotide usage at the third position of a codon and year of virus isolation**. Click here for file

Additional file 13 **Changes in the correlation between codon usage in PB2 and that in human tissue-specific genes over time of viral isolation**. The linear regression line and the correlation coefficient of each dataset are shown. Click here for file
