# Supplementary figures and images for: Codon usage bias and the evolution of influenza A viruses. Codon Usage Biases of Influenza Virus
Source: BMC Evol Biol. 2010 Aug 19;10:253. doi: 10.1186/1471-2148-10-253 (PMC2933640; doi:10.1186/1471-2148-10-253)

## Slide 1
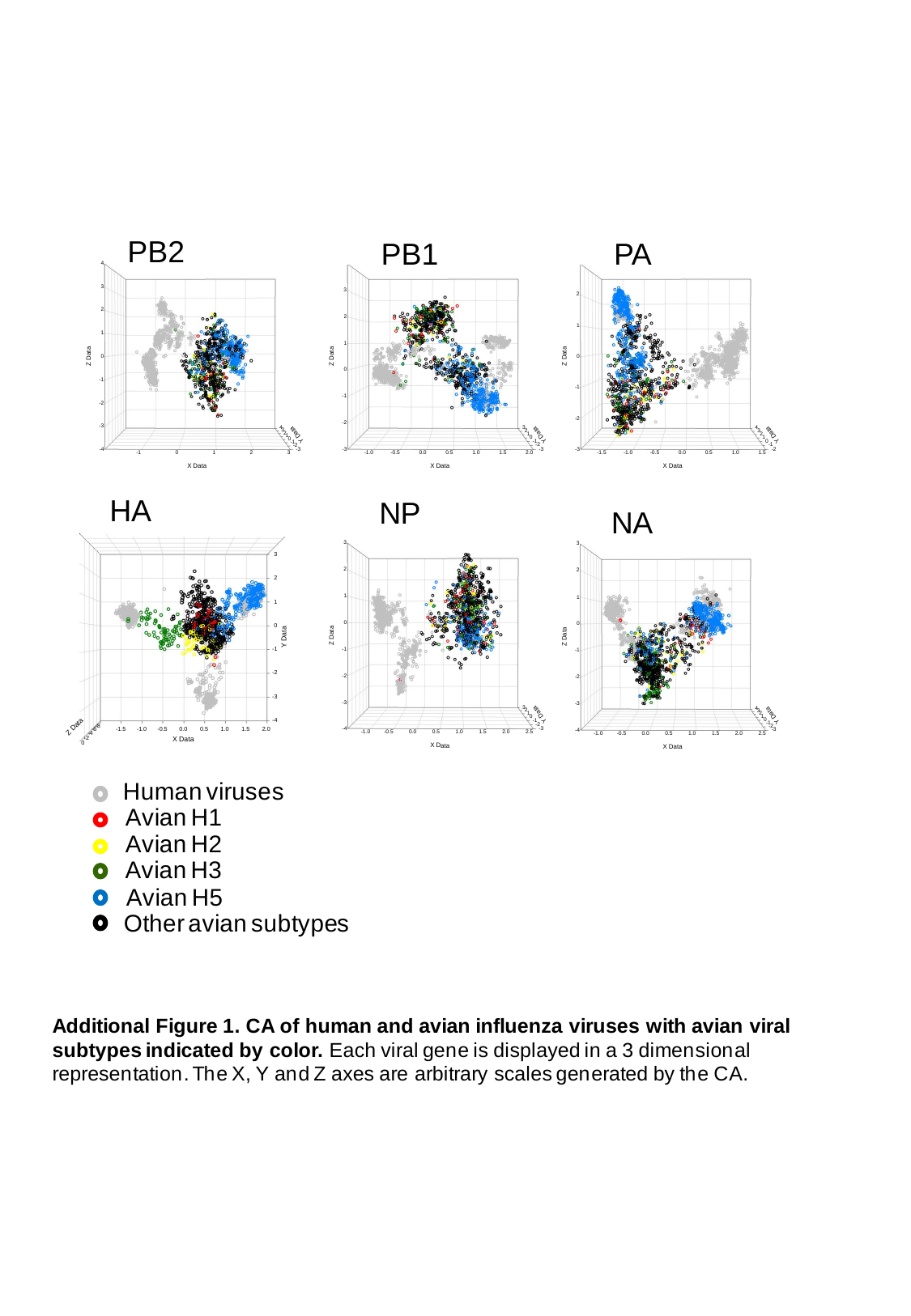

Supplement: Additional file 1 — CA of human and avian influenza viruses with avian viral subtypes indicated by color. Each viral gene is displayed in a 3 dimensional representation. The X, Y and Z axes are arbitrary scales generated by the CA. [file 1471-2148-10-253-S1.PPT]

## Slide 1
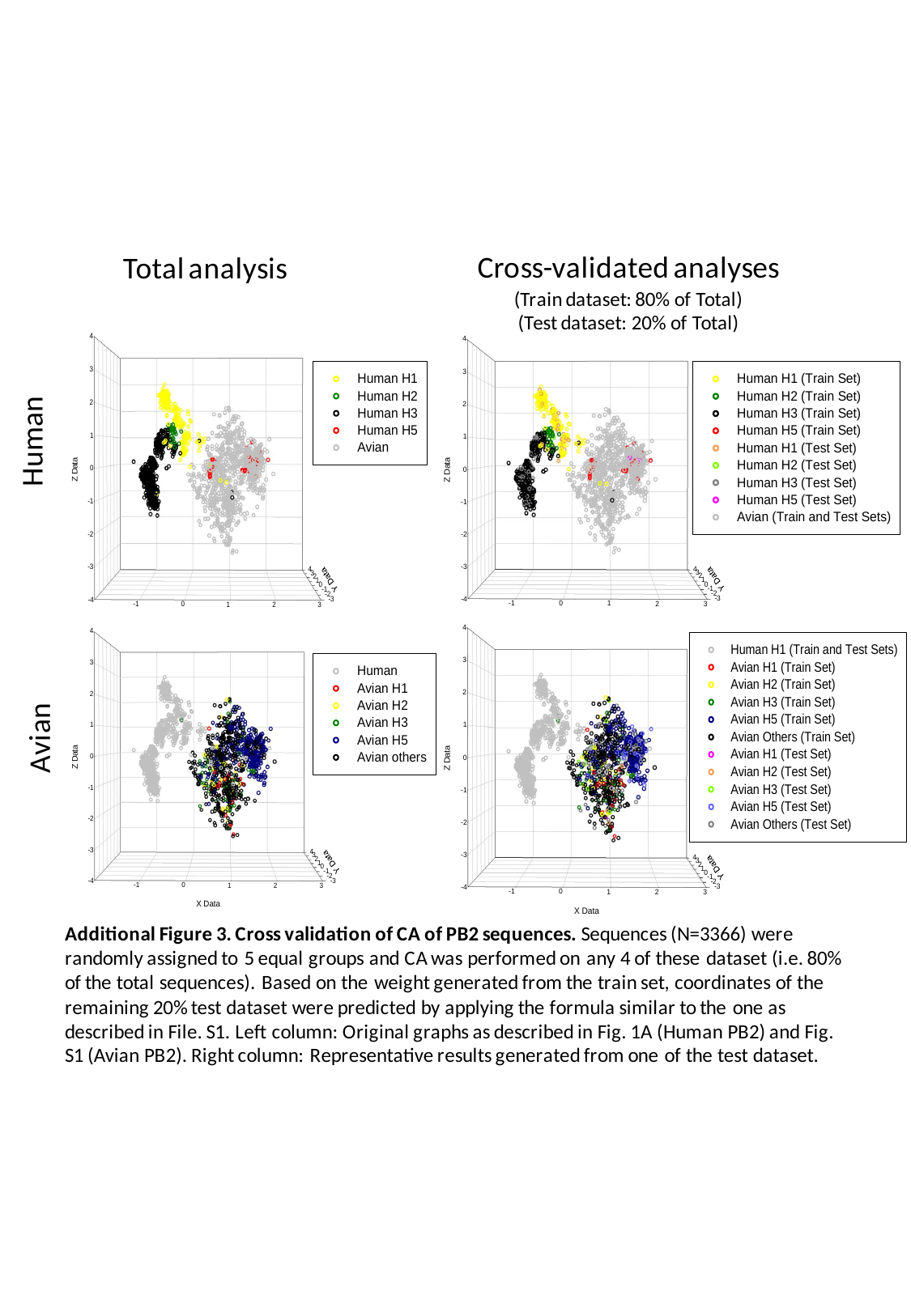

Supplement: Additional file 5 — Cross validation of CA of PB2 sequences. Sequences (N = 3366) were randomly assigned to 5 equal groups and CA was performed on any 4 of these dataset (i.e. 80% of the total sequences). Based on the weight generated from the train set, coordinates of the remaining 20% test dataset were predicted by applying the formula similar to the one as described in Additional file 4. Left column: Original graphs as described in Fig. 1A (Human PB2) and Additional file 1 (Avian PB2). Right column: Representative results generated from one of the test dataset. [file 1471-2148-10-253-S5.PPT]

## Slide 1
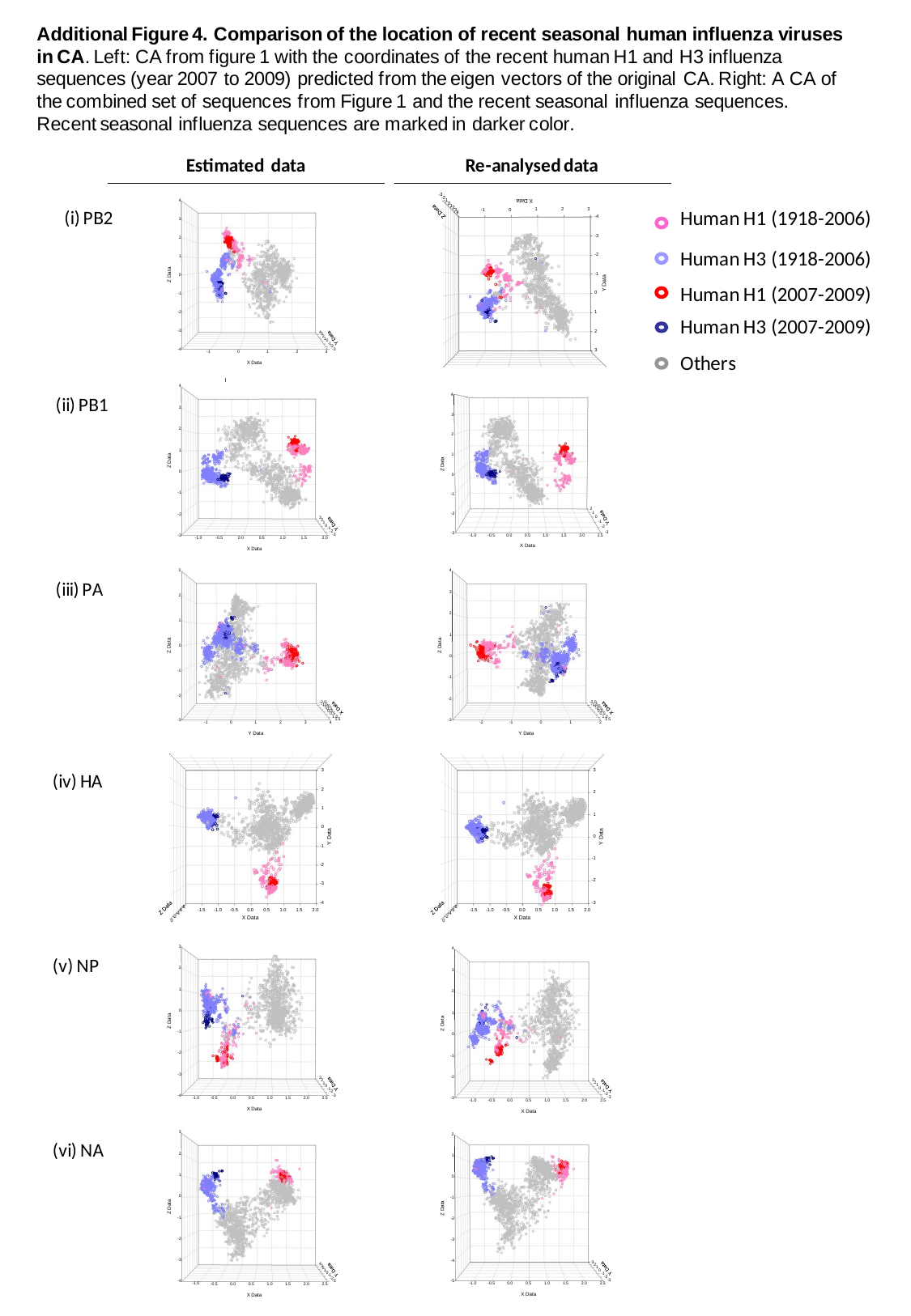

Supplement: Additional file 6 — Comparison of the location of recent seasonal human influenza viruses in CA. Left: CA from figure 1 with the coordinates of the recent human H1 and H3 influenza sequences (year 2007 to 2009) predicted from the eigen vectors of the original CA. Right: A CA of the combined set of sequences from Figure 1 and the recent seasonal influenza sequences. Recent seasonal influenza sequences are marked in darker color. [file 1471-2148-10-253-S6.PPT]

## Slide 1
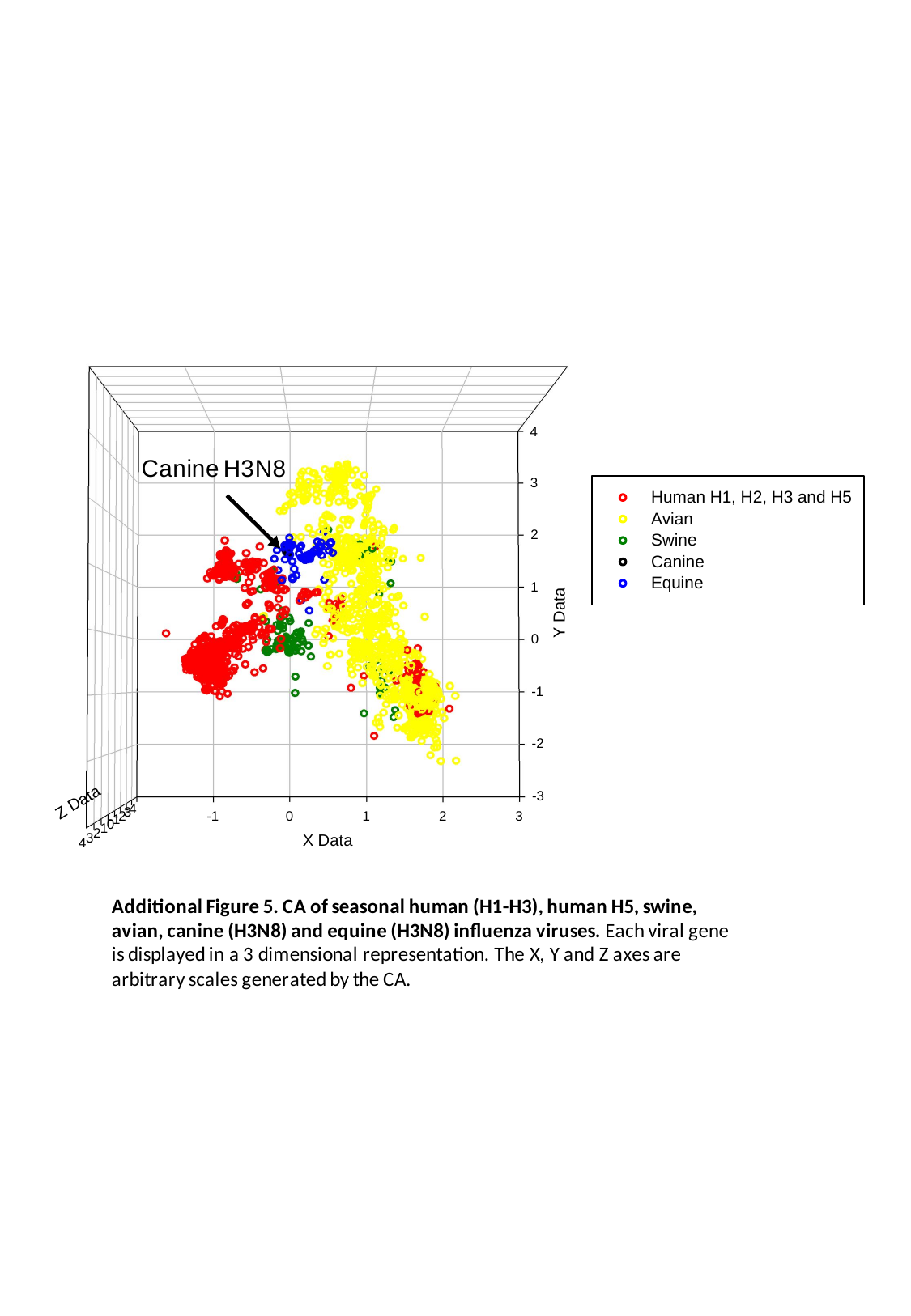

Supplement: Additional file 8 — CA of seasonal human (H1-H3), human H5, swine, avian, canine (H3N8) and equine (H3N8) influenza viruses. Each viral gene is displayed in a 3 dimensional representation. The X, Y and Z axes are arbitrary scales generated by the CA. [file 1471-2148-10-253-S8.PPT]
