# Supplementary material for: Codon usage bias and the evolution of influenza A viruses. Codon Usage Biases of Influenza Virus
Source: BMC Evol Biol. 2010 Aug 19;10:253. doi: 10.1186/1471-2148-10-253 (PMC2933640; doi:10.1186/1471-2148-10-253)
Supplement: Additional file 2 — Outliers are enclosed by open-boxes. Sequence numbers of outliers are indicated (see Additional file 3). Avian virus outliers are marked in red, while human virus outliers are in black. [file 1471-2148-10-253-S2.PPT]

## Slide 1
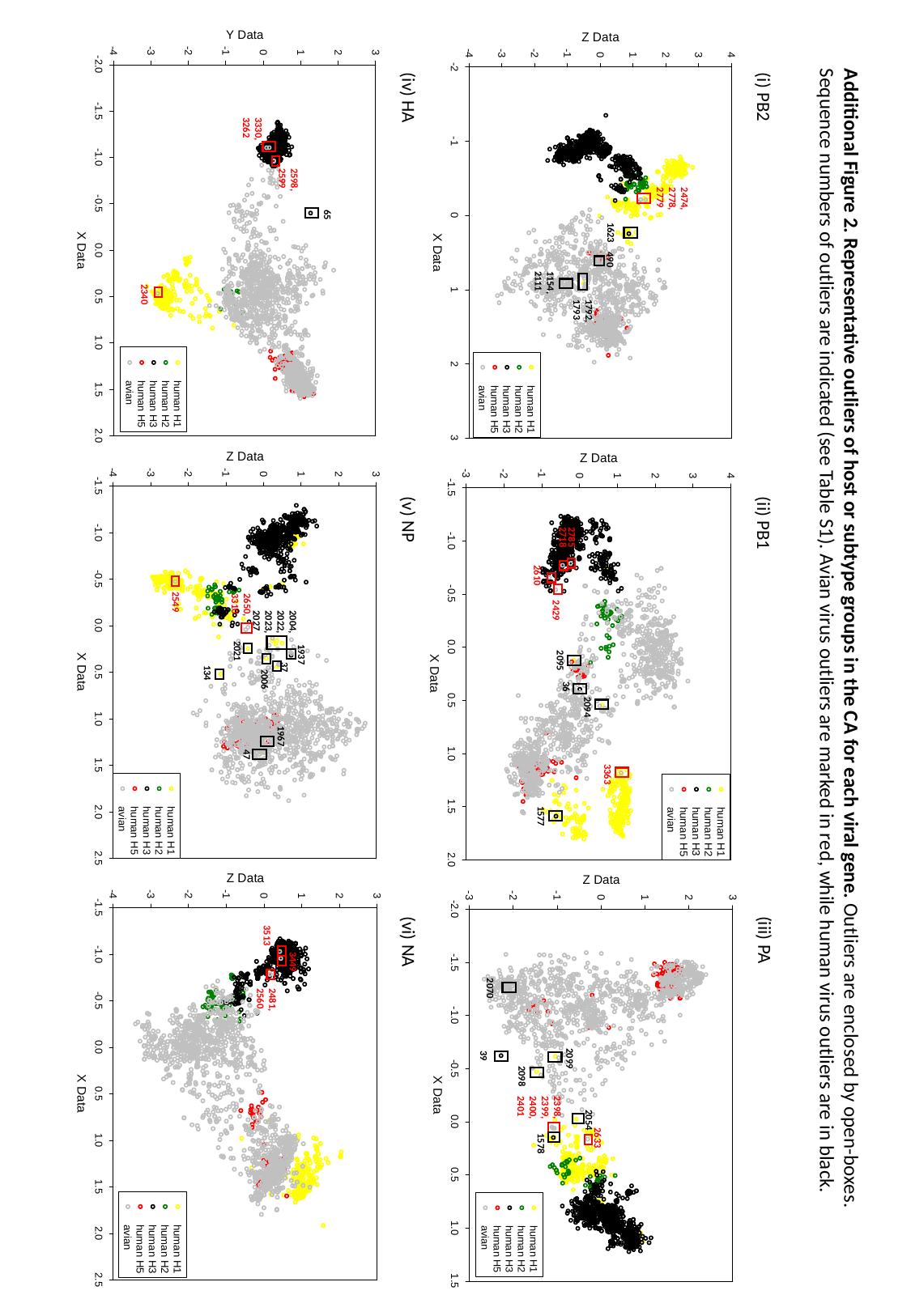

3330,
3262
2598,
2599
 65
2340
2474,
2778,
2779
1623
490
1154,
2111
1792,
1793
(iv) HA
(i) PB2
2650,
3310
2549
2004,
2022,
2023,
2027
2021
1937
134
37
2006
1967
47
2718
2785
2610
2429
2095
36
(iii) PA
2094
3363
1577
(v) NP
(ii) PB1
3513
3449
2481,
2560
2070
 39
2099
2098
2398,
2399,
2400,
2401
2054
2633
1578
(vi) NA
(iii) PA
Additional Figure 2. Representative outliers of host or subtype groups in the CA for each viral gene. Outliers are enclosed by open-boxes. Sequence numbers of outliers are indicated (see Table S1). Avian virus outliers are marked in red, while human virus outliers are in black.
