# Supplementary material for: Codon usage bias and the evolution of influenza A viruses. Codon Usage Biases of Influenza Virus
Source: BMC Evol Biol. 2010 Aug 19;10:253. doi: 10.1186/1471-2148-10-253 (PMC2933640; doi:10.1186/1471-2148-10-253)
Supplement: Additional file 3 — Descriptions of human and avian viral sequences that were marked as outliers in Additional file 2. [file 1471-2148-10-253-S3.DOC]

**Additional Table 1: Descriptions of human and avian viral sequences that were marked as outliers in Additional Figure 2.**

| Segment | host | Seq. no. | Accession | Name | Journal title | Journal details |
| --- | --- | --- | --- | --- | --- | --- |
| PB2 | avian | [2474] | CY005715 | A/red-necked stint/AUS/5745/1981(H12N9) | Large-scale sequence analysis of avian influenza isolates | Science 311 (5767), 1576-1580 (2006) |
|  |  | [2778] | CY014632 | A/red-necked stint/Australia/4189/1980(H4N8) |  |  |
|  |  | [2779] | CY014635 | A/red-necked stint/Australia/4500/1980(H3N8) |  |  |
|  | human | [490] | DQ208309 | A/Brevig Mission/1/1918(H1N1) | Characterization of the 1918 influenza virus polymerase genes | Nature 437 (7060), 889-893 (2005) |
|  |  | [1154] | DQ469955 | A/Ontario/RV1273/2005(H3N2) | Triple Reassortant H3N2 Influenza A Viruses, Canada, 2005 | Emerging Infect. Dis. 12 (7), 1132-1135 (2006) |
|  |  | [1623] | CY015515 | A/Victoria/1968(H3N2) | The NIAID Influenza Genome Sequencing Project | Unpublished |
|  |  | [1792] | EF101747 | A/Philippines/344/2004(H1N2) | Genetic analysis of two influenza A (H1) swine viruses isolated from humans in Thailand and the Philippines | Virus Genes 35 (2), 161-165 (2007) |
|  |  | [1793] | EF101754 | A/Thailand/271/2005(H1N1) |  |  |
|  |  | [2111] | AJ293920 | A/Hong Kong/1774/99(H3N2) | Infection of a child in Hong Kong by an influenza A H3N2 virus closely related to viruses circulating in European pigs | J. Gen. Virol. 82 (PT 6), 1397-1406 (2001) |
| PB1 | avian | [2429] | DQ335777 | A/turkey/Ohio/313053/04(H3N2) | Isolation and characterization of H3N2 influenza A virus from turkeys | Avian Dis. 49 (2), 207-213 (2005) |
|  |  | [2610] | EF551043 | A/turkey/Illinois/2004(H3N2)) segmen | Genetic and Antigenic Relatedness of H3 Subtype Influenza A Viruses Isolated from Avian and Mammalian Species | Unpublished |
|  |  | [2718] | DQ469996 | A/turkey/Ontario/31232/2005(H3N2) | Triple Reassortant H3N2 Influenza A Viruses, Canada, 2005 | Emerging Infect. Dis. 12 (7), 1132-1135 (2006) |
|  |  | [2785] | AY233388 | A/duck/NC/91347/01(H1N2)) polymerase | Characterization of a swine-like reassortant H1N2 influenza virus isolated from a wild duck in the US | Virus Res. 93 (1), 115-121 (2003) |
|  |  | [3363] | CY004120 | A/pintail duck/ALB/628/1979(H6N8) | Large-scale sequence analysis of avian influenza isolates | Science 311 (5767), 1576-1580 (2006) |
|  | human | [36] | 293921:3 | A/Hong Kong/1774/99(H3N2) | Infection of a child in Hong Kong by an influenza A H3N2 virus closely related to viruses circulating in European pigs | J. Gen. Virol. 82 (PT 6), 1397-1406 (2001) |
|  |  | [2094] | EF101748 | A/Philippines/344/2004(H1N2) | Genetic analysis of two influenza A (H1) swine viruses isolated from humans in Thailand and the Philippines | Virus Genes 35 (2), 161-165 (2007) |
|  |  | [2095] | EF101753 | A/Thailand/271/2005(H1N1) |  |  |
|  |  | [1577] | CY015514 | A/Victoria/1968(H3N2) | The NIAID Influenza Genome Sequencing Project | Unpublished |
| PA | avian | [2398] | CY004151 | A/pintail duck/ALB/189/1982(H6N6) | Large-scale sequence analysis of avian influenza isolates | Science 311 (5767), 1576-1580 (2006) |
|  |  | [2399] | CY004159 | A/widgeon/ALB/256/1982(H6N6) |  |  |
|  |  | [2400] | CY004167 | A/blue-winged teal/ALB/266/1982(H6N6) |  |  |
|  |  | [2401] | CY004175 | A/mallard duck/ALB/289/1982(H6N6) |  |  |
|  |  | [2633] | CY005893 | A/pheasant/MN/917/1980(H7N3) |  |  |
|  | human | [39] | 293922:2 | A/Hong Kong/1774/99(H3N2) | Infection of a child in Hong Kong by an influenza A H3N2 virus closely related to viruses circulating in European pigs | J. Gen. Virol. 82 (PT 6), 1397-1406 (2001) |
|  |  | [1578] | CY015513 | A/Victoria/1968(H3N2) | The NIAID Influenza Genome Sequencing Project | Unpublished |
|  |  | [2054] | DQ208311 | A/Brevig Mission/1/1918(H1N1) | Characterization of the 1918 influenza virus polymerase genes | Nature 437 (7060), 889-893 (2005) |
|  |  | [2070] | DQ469957 | A/Ontario/RV1273/2005(H3N2) | Triple Reassortant H3N2 Influenza A Viruses, Canada, 2005 | Emerging Infect. Dis. 12 (7), 1132-1135 (2006) |
|  |  | [2098] | EF101746 | A/Philippines/344/2004(H1N2) | Genetic analysis of two influenza A (H1) swine viruses isolated from humans in Thailand and the Philippines | Virus Genes 35 (2), 161-165 (2007) |
|  |  | [2099] | EF101755 | A/Thailand/271/2005(H1N1) |  | Virus Genes 35 (2), 161-165 (2007) |
| HA | Avian | [2340] | AY180460 | A/Quail/Nanchang/12-340/2000(H1N1) | The influenza virus gene pool in a poultry market in South central china | Virology 305 (2), 267-275 (2003) |
|  |  | [2598] | AY779253 | A/turkey/North Carolina/12344/03(H3N2) | H3N2 Influenza Virus Transmission from Swine to Turkeys, United States | Emerging Infect. Dis. 10 (12), 2156-2160 (2004) |
|  |  | [2599] | AY779254 | A/turkey/Minnesota/764-2/03(H3N2) |  |  |
|  |  | [3262] | DQ335771 | A/turkey/Ohio/313053/04(H3N2) | Isolation and characterization of H3N2 influenza A virus from turkeys | Avian Dis. 49 (2), 207-213 (2005) |
|  |  | [3330] | DQ470002 | A/turkey/Ontario/31232/2005(H3N2) | Triple Reassortant H3N2 Influenza A Viruses, Canada, 2005 | Emerging Infect. Dis. 12 (7), 1132-1135 (2006) |
|  | Human | [65] | AJ293926 | A/Hong Kong/1774/99(H3N2) | Infection of a child in Hong Kong by an influenza A H3N2 virus closely related to viruses circulating in European pigs | J. Gen. Virol. 82 (PT 6), 1397-1406 (2001) |
| NP | avian | [2549] | CY005430 | A/Quail/Nanchang/12-340/2000(H1N1) | Large-scale sequence analysis of avian influenza isolates | Science 311 (5767), 1576-1580 (2006) |
|  |  | [2650] | CY014697 | A/gull/Maryland/704/1977(H13N6) |  |  |
|  |  | [3310] | M27521 | A/gull/Maryland/704/1977(H13N6) | Evolution of the nucleoprotein gene of influenza A virus | J. Virol. 64 (4), 1487-1497 (1990) |
|  | human | [37] | AF342819 | A/Wisconsin/10/98 (H1N1) | Infection of a Wisconsin Man by a Swine-like Influenza Virus containing Avian and Human Polymerase Genes | Unpublished |
|  |  | [47] | AJ293924 | A/Hong Kong/1774/99(H3N2) | Infection of a child in Hong Kong by an influenza A H3N2 virus closely related to viruses circulating in European pigs | J. Gen. Virol. 82 (PT 6), 1397-1406 (2001) |
|  |  | [134] | AY744935 | A/Brevig Mission/1/1918(H1N1) | Novel Origin of the 1918 Pandemic Influenza Virus Nucleoprotein Gene | J. Virol. 78 (22), 12462-12470 (2004) |
|  |  | [1937] | DQ469959 | A/Ontario/RV1273/2005(H3N2) | Triple Reassortant H3N2 Influenza A Viruses, Canada, 2005 | Emerging Infect. Dis. 12 (7), 1132-1135 (2006) |
|  |  | [1967] | EF101752 | A/Thailand/271/2005(H1N1) | Genetic analysis of two influenza A (H1) swine viruses isolated from humans in Thailand and the Philippines | Virus Genes 35 (2), 161-165 (2007) |
|  |  | [2004] | L24394 | A/MD/12/1991(H1N1) | An influenza A (H1N1) virus closely related to swine influenza responsible for a fatal case of human influenza | Unpublished (1993) |
|  |  | [2021] | M63754 | A/New Jersey/8/1976(H1N1) | Evolution of influenza A virus nucleoprotein genes: implications for the origins of H1N1 human and classical swine viruses | J. Virol. 65 (7), 3704-3714 (1991) |
|  |  | [2022] | M63755 | A/Wisconsin/3523/1988(H1N1) |  |  |
|  |  | [2023] | M76602 | A/Ohio/3523/1988(H1N1) | Genetic relatedness of the nucleoprotein (NP) of recent swine, turkey, and human influenza A virus (H1N1) isolates | Virus Res. 22 (1), 79-87 (1992) |
|  |  | [2026] | M76606 | A/New Jersey/8/1976(H1N1) |  |  |
|  |  | [2027] | M76610 | A/Wisconsin/3623/1988(H1N1) |  |  |
| NA | avian | [2481] | AY038015 | A/Turkey/MO/24093/99(H1N2) | Isolation from turkey breeder hens of a reassortant H1N2 influenza virus with swine, human, and avian lineage genes | AvianDis.46(1),111-121(2002) |
|  |  | [2560] | AY233391 | A/duck/NC/91347/01(H1N2) | Characterization of a swine-like reassortant H1N2 influenza virus isolated from a wild duck in the United States | VirusRes.93(1),115-121(2003) |
|  |  | [3449] | DQ335773 | A/turkey/Ohio/313053/04(H3N2) | Isolation and characterization of H3N2 influenza A virus from turkeys | AvianDis.49(2),207-213(2005) |
|  |  | [3513] | DQ470000 | A/turkey/Ontario/31232/2005(H3N2) | Triple Reassortant H3N2 Influenza A Viruses, Canada, 2005 | EmergingInfect.Dis.12(7),1132-1135(2006) |
