# Supplementary material for: Codon usage bias and the evolution of influenza A viruses. Codon Usage Biases of Influenza Virus
Source: BMC Evol Biol. 2010 Aug 19;10:253. doi: 10.1186/1471-2148-10-253 (PMC2933640; doi:10.1186/1471-2148-10-253)
Supplement: Additional file 7 — The ten sequences that were closest to each of the A/Brevig Mission/1/1918 genes. [file 1471-2148-10-253-S7.DOC]

**Additional Table 2.** The ten sequences that were closest to each of the A/Brevig Mission/1/1918 genes.

| Segment | Host | Accession number | Subtype | Year | Name | Remark |
| --- | --- | --- | --- | --- | --- | --- |
| PB2 | Avian | AF508659 | H9N2 | 1997 | A/Chicken/Shenzhen/9/97 |  |
|  | Avian | DQ064551 | H9N2 | 1997 | A/chicken/Guangdong/6/97 |  |
|  | Avian | DQ366303:28-2307 | H5N1 | 2005 | A/duck/Vietnam/1/2005 |  |
|  | Swine | AB434349 | H3N2 | 2004 | A/swine/Udon Thani/NIAH464/2004 |  |
|  | Avian | AF156432 | H9N2 | 1997 | A/Pigeon/Hong Kong/Y233/97 |  |
|  | Human | AF258839:28-2307 | H5N1 | 1997 | A/Hong Kong/483/97 | Avian-origin H5N1 |
|  | Avian | DQ064569 | H9N2 | 1997 | A/chicken/Shenzhen/9/97 |  |
|  | Avian | AF156431 | H9N2 | 1997 | A/Chicken/Hong Kong/G23/97 |  |
|  | Human | AF084262:1-2280 | H5N1 | 1997 | A/HongKong/483/97 | Avian-origin H5N1 |
|  | Avian | DQ064552 | H9N2 | 1999 | A/chicken/Guangxi/10/99 |  |
|  |  |  |  |  |  |  |
| PB1 | Swine | M55472 | H1N1 | 1931 | A/swine/1976/1931 |  |
|  | Swine | CY022403 | H1N1 | 1975 | A/swine/Tennessee/1/1975 |  |
|  | Swine | CY009634 | H1N1 | 1931 | A/swine/1931 |  |
|  | Swine | CY026433 | H1N1 | 1942 | A/swine/Jamesburg/1942 |  |
|  | Swine | CY027297 | H1N1 | 1935 | A/swine/Ohio/23/1935 |  |
|  | Swine | CY022043 | H1N1 | 1976 | A/swine/Tennessee/7/1976 |  |
|  | Swine | CY022035 | H1N1 | 1976 | A/swine/Tennessee/3/1976 |  |
|  | Swine | CY026297 | H1N1 | 1967 | A/swine/Wisconsin/1/1967 |  |
|  | Swine | M25931 | H1N1 | 1977 | A/swine/Tennessee/26/1977 |  |
|  | Swine | CY009922 | H1N1 | 1977 | A/swine/Tennessee/25/1977 |  |
|  |  |  |  |  |  |  |
| PA | Avian | CY004767 | H4N6 | 1977 | A/pintail duck/ALB/159/1977 |  |
|  | Avian | CY004183 | H6N4 | 1982 | A/blue-winged teal/ALB/685/1982 |  |
|  | Swine | CY026432 | H1N1 | 1942 | A/swine/Jamesburg/1942 |  |
|  | Swine | M55473 | H1N1 | 1931 | A/swine/1976/1931 |  |
|  | Avian | CY014736 | H1N1 | 1981 | A/duck/Minnesota/1375/1981 |  |
|  | Avian | CY014942 | H4N3 | 1982 | A/pintail duck/New York/155/1982 |  |
|  | Avian | CY005871 | H1N1 | 1981 | A/pigeon/MN/1407/1981 |  |
|  | Avian | CY004479 | H1N1 | 1977 | A/pintail duck/ALB/219/1977 |  |
|  | Avian | CY004754 | H4N3 | 1977 | A/mallard/Alberta/300/1977 |  |
|  | Avian | CY005713 | H12N9 | 1981 | A/red-necked stint/AUS/5745/1981 |  |
|  |  |  |  |  |  |  |
| HA | Human | DQ508905 | H1N1 | 1933 | A/Wilson-Smith/1933 |  |
|  | Swine | CY026427 | H1N1 | 1942 | A/swine/Jamesburg/1942 |  |
|  | Human | J02176 | H1N1 | 1933 | A/WSN/33 |  |
|  | Swine | CY027291 | H1N1 | 1935 | A/swine/Ohio/23/1935 |  |
|  | Human | U37727 | H1N9 | 1933 | A/NWS/G70C |  |
|  | Human | CY009604 | H1N1 | 1933 | A/Wilson-Smith/33 |  |
|  | Human | U38242 | H1 | 1967 | A/Tokyo/3/1967 |  |
|  | Swine | D00837 | H1N1 | 1939 | A/swine/Cambridge/1939 |  |
|  | Human | CY010788 | H1N1 | 1933 | A/WSN/1933 TS61 |  |
|  | Human | AF408859 | H1N9 | 1933 | A/NWS-G70c |  |
|  |  |  |  |  |  |  |
| NP | Avian | CY003987 | H2N4 | 2002 | A/mallard/Alberta/149/2002 |  |
|  | Avian | DQ021808 | H6N8 | 2002 | A/wild bird/DE/1185/02 |  |
|  | Avian | AF098626 | H5N2 | 1994 | A/Chicken/Puebla/8623-607/94 |  |
|  | Avian | AF098627 | H5N2 | 1994 | A/Chicken/Puebla/14585-622/94 |  |
|  | Avian | CY004557 | H2N8 | 1988 | A/herring gull/DE/692/1988 |  |
|  | Avian | CY004091 | H6N8 | 1988 | A/knot/DE/526/1988 |  |
|  | Avian | CY003910 | H2N1 | 1988 | A/herring gull/DE/698/1988 |  |
|  | Avian | CY003917 | H2N8 | 1988 | A/herring gull/DE/703/1988 |  |
|  | Avian | CY005300 | H11N1 | 1989 | A/ruddy turnstone/NJ/842/1989 |  |
|  | Avian | CY014557 | H2N9 | 1988 | A/herring gull/Delaware/670/1988 |  |
|  |  |  |  |  |  |  |
| NA | Swine | EU139834 | H1N1 | 1945 | A/swine/Iowa/1945 |  |
|  | Swine | AF250364 | H1N1 | 1930 | A/Swine/Iowa/30 |  |
|  | Swine | CY009630 | H1N1 | 1931 | A/swine/1931 |  |
|  | Swine | EU139833 | H1N1 | 1930 | A/swine/Iowa/15/1930 |  |
|  | Swine | CY026429 | H1N1 | 1942 | A/swine/Jamesburg/1942 |  |
|  | Swine | CY027293 | H1N1 | 1935 | A/swine/Ohio/23/1935 |  |
|  | Human | AJ518097 | H1N1 | 1995 | A/Wuhan/371/95 |  |
|  | Human | CY013815 | H1N1 | 1996 | A/Nanchang/13/1996 |  |
|  | Human | CY013823 | H1N1 | 1996 | A/Nanchang/15/1996 |  |
|  | Human | CY013831 | H1N1 | 1996 | A/Nanchang/16/1996 |  |

Remark: Sequences were ranked according to their distances to A/Brevig Mission/1/1918 in ascending order.
