# Supplementary material for: Codon usage bias and the evolution of influenza A viruses. Codon Usage Biases of Influenza Virus
Source: BMC Evol Biol. 2010 Aug 19;10:253. doi: 10.1186/1471-2148-10-253 (PMC2933640; doi:10.1186/1471-2148-10-253)
Supplement: Additional file 9 — Overall codon usage of Influenza virus types and their hosts. Under-represented codons (RSCU < 0.6) are highlighted in grey, while the most commonly used codons are in bold. [file 1471-2148-10-253-S9.DOC]

**Additional Table 3.** **Overall codon usage of Influenza virus types and their hosts.** Under-represented codons (RSCU<0.6) are highlighted in grey, while the most commonly used codons are in bold.
