# Supplementary material for: Codon usage bias and the evolution of influenza A viruses. Codon Usage Biases of Influenza Virus
Source: BMC Evol Biol. 2010 Aug 19;10:253. doi: 10.1186/1471-2148-10-253 (PMC2933640; doi:10.1186/1471-2148-10-253)
Supplement: Additional file 10 — Codons with positive (R ≥ 0.5) and negative (R ≤ -0.5) correlations in codon usage over time of viral isolation in human H1N1, human H3N2 and avian influenza viruses. [file 1471-2148-10-253-S10.DOC]

**Additional Table 4. Codons with positive (R≥0.5) and negative (R≤-0.5) correlations in codon usage over time of viral isolation in human H1N1, human H3N2 and avian influenza viruses.**
