# Supplementary material for: Codon usage bias and the evolution of influenza A viruses. Codon Usage Biases of Influenza Virus
Source: BMC Evol Biol. 2010 Aug 19;10:253. doi: 10.1186/1471-2148-10-253 (PMC2933640; doi:10.1186/1471-2148-10-253)
Supplement: Additional file 11 — Correlation coefficient (R) between viral GC content and year of virus isolation. [file 1471-2148-10-253-S11.DOC]

**Additional Table** 5. Correlation coefficient (R) between viral GC content and year of virus isolation.

|  | PB2 | PB1 | PA | HA | NP | NA |
| --- | --- | --- | --- | --- | --- | --- |
| Human H1N1 | -0.83 | -0.70 | -0.63 | 0.06 | -0.50 | -0.85 |
| Human H3N2 | -0.59 | -0.18 | -0.29 | -0.89 | -0.65 | -0.15 |
| Avian | -0.23 | -0.07 | -0.21 | -0.35 | -0.13 | 0.09 |

Remarks: R<-0.5 are highlighted.
