# Supplementary material for: Codon usage bias and the evolution of influenza A viruses. Codon Usage Biases of Influenza Virus
Source: BMC Evol Biol. 2010 Aug 19;10:253. doi: 10.1186/1471-2148-10-253 (PMC2933640; doi:10.1186/1471-2148-10-253)
Supplement: Additional file 12 — Correlation coefficient (R) between nucleotide usage at the third position of a codon and year of virus isolation. [file 1471-2148-10-253-S12.DOC]

**Table S6. Correlation coefficient(R) between nucleotide usage at the third position of a codon and year of virus isolation.**

| Virus | 3' nucleotide | PB2 | PB1 | PA | HA | NP | NA |
| --- | --- | --- | --- | --- | --- | --- | --- |
| Human | 3' G | -0.89 | -0.43 | 0.13 | -0.50 | -0.31 | -0.81 |
| H1N1 | 3' T | 0.33 | 0.48 | 0.37 | 0.60 | -0.01 | -0.33 |
|  | 3' C | -0.24 | -0.38 | -0.83 | -0.14 | -0.21 | 0.43 |
|  | 3' A | 0.88 | 0.12 | 0.50 | -0.36 | 0.46 | 0.84 |
| Human | 3' G | -0.77 | 0.20 | -0.35 | -0.76 | 0.41 | -0.42 |
| H3N2 | 3' T | -0.05 | -0.43 | -0.54 | 0.23 | 0.27 | -0.73 |
|  | 3' C | 0.46 | 0.22 | 0.60 | -0.47 | -0.78 | 0.61 |
|  | 3' A | 0.68 | 0.02 | 0.33 | 0.88 | 0.50 | 0.52 |
| Avian | 3' G | -0.11 | 0.09 | -0.40 | 0.08 | 0.01 | -0.07 |
|  | 3' T | 0.39 | 0.29 | -0.14 | -0.23 | -0.10 | 0.30 |
|  | 3' C | -0.33 | -0.24 | 0.05 | -0.04 | -0.29 | -0.32 |
|  | 3' A | 0.03 | -0.31 | 0.38 | 0.19 | 0.14 | -0.43 |

Remarks: R with a value <-0.5 or >0.5 is highlighted in red or green, respectively.
