# Supplementary material for: Codon usage bias and the evolution of influenza A viruses. Codon Usage Biases of Influenza Virus
Source: BMC Evol Biol. 2010 Aug 19;10:253. doi: 10.1186/1471-2148-10-253 (PMC2933640; doi:10.1186/1471-2148-10-253)
Supplement: Additional file 13 — Changes in the correlation between codon usage in PB2 and that in human tissue-specific genes over time of viral isolation. The linear regression line and the correlation coefficient of each dataset are shown. [file 1471-2148-10-253-S13.PPT]

## Slide 1
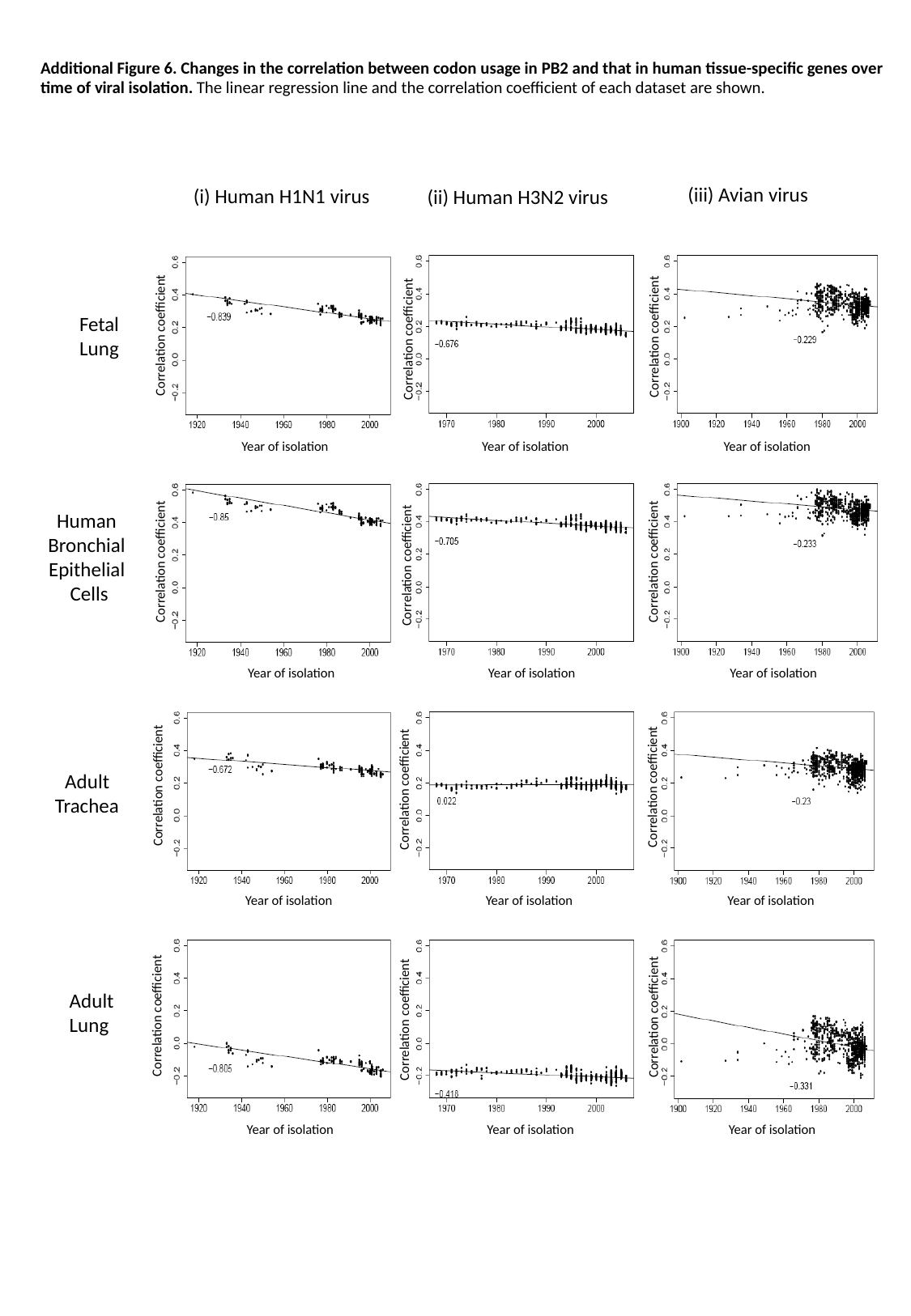

Additional Figure 6. Changes in the correlation between codon usage in PB2 and that in human tissue-specific genes over time of viral isolation. The linear regression line and the correlation coefficient of each dataset are shown.
(iii) Avian virus
(i) Human H1N1 virus
(ii) Human H3N2 virus
Correlation coefficient
Correlation coefficient
Correlation coefficient
Correlation coefficient
Correlation coefficient
Correlation coefficient
Correlation coefficient
Correlation coefficient
Correlation coefficient
Correlation coefficient
Correlation coefficient
Correlation coefficient
Fetal
Lung
Year of isolation
Year of isolation
Year of isolation
Human
Bronchial
Epithelial
Cells
Year of isolation
Year of isolation
Year of isolation
Adult
Trachea
Year of isolation
Year of isolation
Year of isolation
Adult
Lung
Year of isolation
Year of isolation
Year of isolation
